# What if implant-based prosthodontics would have a standard screw head?

**DOI:** 10.1186/s40729-025-00651-5

**Published:** 2025-10-02

**Authors:** Stefano Pieralli, Florian Beuer, Michael Naumann

**Affiliations:** 1 Department of Prosthodontics, Geriatric Dentistry and Craniomandibular Disorders, Charité – Universitätsmedizin Berlin, Aßmannshauser Straße 4-6, 14197 Berlin, Germany; 2 Private dental office, Berlin, Germany

## Abstract

The management of a loosened screw of an implant-based restoration – whether or not documentation of the respective implant system is available – is a common and challenging complication in dental practice simply due to the fact that the right screw driver must be available. This short communication highlights the need for technical standardization of certain components in implant prosthodontics. For example, screw head geometries, show a wide range between the systems and manufacturers, and make it necessary to provide matching screw drivers. Subsequently, prosthetic maintenance becomes less time and cost effective. Comparisons to the consumer electronics industry are drawn, where standardization improves usability and sustainability. It suggests that voluntary cooperation within the industry could create a unified standard. Standardization would facilitate clinical workflows in particular when complications occur, enhance patient safety, reduce costs, and improve long-term outcomes of implant-based restorations.

Imagine a Monday morning, a new patient presents in your dental office with a mobile implant crown. You miss the information about the implant system or the right screw driver is just not in the office, which is a very likely and common situation given the large variety of implant systems on the market. Screw loosening represents one of the most frequent technical complications in implant prosthodontics, particularly in the case of single-unit restorations [1, 2]. Its etiology is multifactorial, including factors as occlusal overload, and inadequate torque application [3]. However, intraoral “normal” dynamic loading over time as such is likely to cause loosening as it is typical when two technical components are linked to each other. This technical complication can result in gap formation and subsequent microleakage at the implant–abutment interface. This again may cause adverse effects on both peri-implant soft and hard tissues, i.e. peri-implant bone loss [4]. Finally, persistent screw loosening may ultimately lead to a fracture of the screw and linked components as a ceramic abutment. Screw fracture is considered as relatively complex type of complication, since fractured segments might remain blocked within the screw channel. The retrieval of the fractured fragment requires specific and delicate procedures, due to the risk of damaging the implant–abutment connection or the internal configuration of the implant itself [5].

The presented exemplary case involves an implant-supported crown in the region of the maxillary central incisor and installed several years before. The patient is unaware of the implant system used, has no implant passport or related documentation, and the original treating dentist has retired. Now, as the clinician managing the case, you’re confronted with a time-sensitive issue in a highly visible esthetic zone. You begin with a clinical examination and by taking, when needed, a radiograph in the hope of identifying the implant system using personal experience or AI-based diagnostic software [6–8].

However, identifying the implant is only the first of two significant hurdles. The second—and often more disruptive—challenge is mechanical access. The screw securing the crown requires a specific driver, one that may not be part of the clinician’s existing armamentarium. In fact, considering the vast number of implant systems on the market and the variety of proprietary screw head geometries, it is statistically unlikely that the correct driver is always immediately available. In most of the cases, clinicians must either order the original driver from the implant manufacturer or attempt to find a functional equivalent in a universal driver kit. Both approaches are inefficient and often delay the treatment, extending the clinical appointment, and increase its costs. Such inefficiencies can create significant burdens for both patients and clinicians, especially in cases where urgent intervention – as in the esthetic zone of an upper central incisor is needed.

Analogies to other technical fields are instructive. On one side, in international travel, plug adapters are an accepted but inconvenient workaround for incompatible power outlets. On the other side, in consumer electronics, the proliferation of proprietary charging cables was a longstanding source of user frustration until regulatory intervention prompted the adoption of USB-C as a universal standard. For example, the European Union’s mandate for a common charger, which applies to mobile phones, tablets, laptops, and other small devices [9], was celebrated as a victory for consumer rights and sustainability. Therefore, by similar reasoning, this approach could be applied to healthcare devices, particularly those that are surgically implanted in the human body and may require servicing or revision years or even decades later. This concept is already exemplified by cardiac devices such as defibrillators, where established standards have proven beneficial for long-term maintenance by facilitating the interchangeability of components [10].

Despite all the clinical rationale for compatibility, there are several challenges to standardization. Manufacturers create proprietary screw geometries (I) to protect their intellectual property, (II) to build brand loyalty, and (III) keep clinicians within their ecosystem. Additionally, (IV) it might be seen as a strategy to maintain market share and offer system exclusivity. Furthermore, the international dental implant industry is highly fragmented, with over 150 manufacturers (offering one or more implant systems) worldwide with considerable variation in market value and penetration across regions [11]. Unlike consumer electronics, which are governed by international directives, there is no top-down regulation in implant dentistry yet. While some third-party companies have introduced kits featuring a wide range of driver geometries, data on their clinical performance remain sparse [12, 13].

The current situation highlights a clear tension between innovation and need for interoperability. Though proprietary design has enabled immense advances in implant technology, it has also introduced unnecessary obstacles for clinicians during treatment. A technically sound and ethically desirable step forward would be for major implant systems to voluntarily adopt standardized screw-head designs. This development doesn’t have to come through strict regulations—instead, research and collaboration should focus on finding a standard that achieves the right balance between clinical effectiveness and design quality.

We understand this manuscript as discussion contribution to a “*Save Implant Life Initiative*”, to promote long-term serviceability of implants and implant-supported prostheses. Clinicians should be provided with reliable, immediate accessible, compatible instruments, regardless of the implant’s origin or age. Such standardization would increase clinical efficiency, reduce unnecessary interventions and enhance patient safety and high-quality care without complications. As implant dentistry progresses, we face a choice: to persist with fragmented proprietary solutions or to move toward shared technical standards that would reflect both scientific responsibility and practical necessity for the benefit of the patient.

## Data Availability

No datasets were generated or analysed during the current study.
